# Is adjuvant chemotherapy necessary in pT1N1 gastric cancer?

**DOI:** 10.1186/s12885-017-3265-x

**Published:** 2017-04-22

**Authors:** Hyun Beak Shin, Ji Yeong An, Seung Hyoung Lee, Yoon Young Choi, Jong Won Kim, Soo Sang Sohn, Sung Hoon Noh

**Affiliations:** 1 Department of Surgery, Chonbuk National University Medicine School, Jeonju, Korea; 2 Department of Surgery, Samsung Medical Center, Sungkyunkwan University School of Medicine, Seoul, Korea; 3 Department of Surgery, Keimyung University School of Medicine, Daegu, Korea; 4 Department of Surgery, Yonsei University Health System, Yonsei University College of Medicine, 134, Shinchon-dong, Seodaemun-gu, Seoul, Korea

**Keywords:** pT1N1, Gastric cancer, Adjuvant chemotherapy, Recurrence, Treatment strategy

## Abstract

**Background:**

Due to a lack of consensus on adjuvant treatments for pT1N1 gastric cancer, surgeons face a dilemma when deciding treatments for patients with pT1N1 gastric cancer after gastrectomy. The objective of this study was to determine survival benefits of adjuvant chemotherapy and risk factors for tumor recurrence in gastric cancer patients with pT1N1.

**Methods:**

Between 1996 and 2010, 510 patients who underwent curative resection for pT1N1 gastric cancer at three institutes were divided into two groups: adjuvant chemotherapy group (*N* = 150) and surgery-only group (*N* = 360). Disease-free survival rates and risk factors for tumor recurrence were analyzed.

**Results:**

During the median follow-up of 78 months, 7.5% of patients experienced tumor recurrence (7.3% in adjuvant chemotherapy group and 7.5% in surgery-only group). The 5-year disease-free survival rate was 91.8% in the adjuvant chemotherapy group and 94.6% in the surgery-only group without significant difference between the two. In univariate analysis, older age (>65 years), male gender, body mass index <25 kg/m^2^, elevated gross type, and differentiated histology were associated with tumor recurrence. Multivariate analysis showed that advanced age and male gender were independent risk factors for tumor recurrence. In addition, adjuvant chemotherapy showed no benefitial effect on tumor recurrence in pT1N1 gastric cancer.

**Conclusions:**

Adjuvant chemotherapy did not show any oncologically benefitial effect on tumor recurrence, it might be unnecessary for pT1N1 gastric cancer after curative surgery.

## Background

According to the National Comprehensive Cancer Network (NCCN) Guidelines (Version 2.2013, Gastric Cancer), treatment for patients with pT1N1 gastric cancer confined to the mucosa or submucosa with one or two regional lymph node metastases should include adjuvant chemotherapy after curative resection [1, 2]. Meanwhile, the Japanese Gastric Cancer Treatment Guidelines 2010 (Version 3) recommend observation without adjuvant treatment after curative resection for patinets with pathologic stage I (including pT1N1) gastric cancer [3]. Since the two representative treatment guidelines present inconsistent views on this issue, adding adjuvant chemotherapy after surgical treatment in pT1N1 gastric cancer might depend on policies or preferences of individual institutions, surgeons, and oncologists. Although NCCN guidelines recommend adjuvant chemotherapy which contains 5-FU ± leucovorin or capecitabine, then fluoropyrimidiene-based chemoradiation, then 5-FU ± leucovorin or capecitabine (Category1) and chemotherapy for patients who have undergone primary D2 lymph node dissection for any T, N + (including pT1N1) gastric cancer [4], uncertainty on the benefit of adjuvant chemotherapy and excellent prognosis of pT1N1 gastric cancer make decisions more difficult in clinical practice.

Although the prognosis of early gastric cancer (EGC) is excellent with a 5-year survival rate after curative resection of more than 90%, recurrence occurs in approximately 1.4–7.0% of patients [5]. Several clinicopathological factors such as lymph node metastasis, elevated gross type, lymphovascular invasion, perineural invasion, a ratio of metastatic-to-retrieved lymph nodes of greater than 0.07, advanced age, and differentiated histologic type have been reported as predictive factors for tumor recurrence in EGC [6–18]. Lymph node metastasis is the most important prognostic factor of tumor recurrence after curative resection in EGC. A higher rate of recurrence is expected for pT1N1 than for pT1N0 gastric cancer. This might be the basis of NCCN guidelines. However, studies have yet to evaluate tumor recurrence, prognostic factors, or the survival benefit of adjuvant chemotherapy for pT1N1 gastric cancer. Moreover, there is no consensus on adjuvant chemotherapy for pT1N1 gastric cancer.

Therefore, the objective of this study was to evaluate the oncological benefit of adjuvant chemotherapy after curative surgery and identify factors predictive of tumor recurrence for pT1N1 gastric cancer.

## Methods

### Patients

Clinicopathological data of 510 gastric cancer patients who had undergone gastrectomy with lymph node dissection and finally diagnosed with pT1N1 between 1996 and 2010 at three institutions (Severance Hospital, Gangnam Severance Hospital, and Dongsan Medical Center) were reviewed. Patients with prior gastric surgery, double primary malignancies, and neoadjuvant chemotherapy were excluded. This study was reviewed and approved by the Institutional Review Boards of Severance Hospital, Yonsei University College of Medicine (4–2014-0317) and Dongsan Medical Center, Keimyung University School of Medicine (2014–08-005).

### Evaluation of clinicopathological variables

Clinicopathological features included for analysis in the present study comprised age, sex, BMI, resection extent, tumor location, gross type, histological type, number of metastatic lymph nodes, adjuvant chemotherapy, and tumor recurrence based on information listed in a prospectively designed database. Conventional open gastrectomy was performed in 422 patients. Minimally invasive surgery including laparoscopic and robot-assisted gastrectomy was performed in 88 patients. In all patients, subtotal or total gastrectomy with D1+ or D2 lymph node dissection was performed according to the guidelines of the Japanese Gastric Cancer Association [3]. Histological types were divided into differentiated type (including papillary adenocarcinoma and well-to-moderately differentiated tubular adenocarcinoma) and undifferentiated type (including poorly differentiated tubular adenocarcinoma, mucinous adenocarcinoma, and signet ring cell adenocarcinoma). Gross classification for EGC followed the Japanese Endoscopy Society Classification. Gross types were divided into elevated type (including EGC gross types I and IIa) and non-elevated type (including EGC gross types IIb, IIc, and III) [19]. For most patients, 5-FU-based oral chemotherapeutic agents including Tegafur/uracil (UFT), Furtulon, Titanium silicate (TS)-1, Tegacil, Didox, and Mifurol were used. A few intravenous regimens (5-fluorouracil + adriamycin, 5-fluorouracil + Cis-Dichloro-Diamine-Platinum, mitomycin-C + 5-fluorouracil +Cytarabine, and 5-fluorouracil + adriamycin + mitomycin-C) were used in some patients for adjuvant chemotherapy. Since there is no established treatment strategy for pT1N1 gastric cancer, decisions to administer chemotherapy in those patients was based on their surgeons’ or oncologists’ preference. The prognostic effect of adjuvant chemotherapy was evaluated by comparing disease-free survival of patients with chemotherapy to those without chemotherapy. Tumor recurrence was identified according to standard clinical practices by evaluating patients every 3 or 6 months until 2 years after surgery and then every 6 months thereafter for up to 5 years after surgery with physical examinations, laboratory tests, imaging (abdomen-pelvis CT and chest X-ray), and endoscopy. Confirmation of recurrence by tissue biopsy was done when possible. Liver magnetic resonance imaging, bone scans, and positron emission tomography scans were optional. The patterns of tumor recurrence were classified as remnant stomach, peritoneal, hematogenous, distant lymph node, and mixed type. Recurrence in remnant stomach included tumor reappearance in the remnant stomach or anastomotic site. Peritoneal seeding and Krukenberg tumor were considered as peritoneal recurrence. Hematogenous spread was defined when there was involvement of specific intra-abdominal or extra-abdominal organs such as liver, lung, bone, brain, or adrenal glands. Recurrence in distant lymph nodes included paraaortic, aortocaval, retroperitoneal, retropancreatic, and extra-abdominal lymph node metastasis. Mixed type metastasis was considered when cases had more than two types of metastasis.

### Statistical analysis

Statistical analysis was carried out using SPSS® version 20.0 for Windows® (SPSS, Chicago, Illinois, USA). Categorical variables were compared using Chi-square or Fisher’s exact tests. Univariate and multivariate analyses for factors predictive of tumor recurrence after curative resection for pT1N1 gastric cancer were carried out using Cox proportional hazards model. Disease-free survival curves were analyzed using the Kaplan-Meier method with the duration of disease-free survival calculated in months based on the length of time between primary surgical treatment and the last follow-up or recurrence. *P* values <0.05 were considered statistically significant.

## Results

### Effect of adjuvant chemotherapy on disease-free survival

**Fig. 1:**
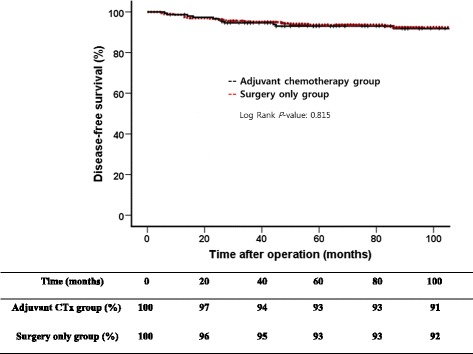
Comparison of disease-free survivals between adjuvant chemotherapy group and surgery-only group

Among a total of 510 patients, 150 (29.4%) patients received adjuvant chemotherapy after curative gastrectomy and 360 (70.6%) patients underwent surgery only. Clinicopathological characteristics of the adjuvant chemotherapy group and the surgery only group are summarized in Table 1. The adjuvant chemotherapy group showed higher incidence of two metastatic lymph nodes (43.3% vs. 31.9%) and lymphovascular invasion (60.7% vs. 41.7%) compared to the surgery-only group. However, there was no difference in disease-free survival between patients with one metastatic lymph node and those with two metastatic lymph nodes. There was no difference in disease-free survial between patients with lymphovascular invasion and those without lymphovascular invasion either. Five year disease-free survival rate was 91.8% in the adjuvant chemotherapy group and 94.6% in the surgery only group without significant difference (*P* = 0.815) (Fig. 1).

**Table 1:** Comparison of clinicopathological characteristics between adjuvant chemotherapy group and surgery-only group

	Adjuvant chemotherapy (*N* = 150)		Surgery-only (*N* = 360)		*P*-value
	*N*	%	*N*	%	
Age (years)					
≤ 65	108	72.0	246	69.3	0.461
> 65	42	28.0	114	31.7	
Gender					0.920
Male	95	63.3	225	62.5	
Female	55	36.7	135	37.5	
BMI (kg/m2)					0.990
< 25	102	68.0	245	68.1	
≥ 25	48	32.0	115	31.9	
Type of resection					0.060
STG	140	93.3	315	87.5	
TG	10	6.7	45	12.5	
Reconstruction					0.168
BI	70	46.7	174	48.3	
BII	68	45.3	139	38.6	
Roux-en-Y	12	8.0	47	13.1	
Tumor location					0.416
Upper	9	6.0	20	5.6	
Middle	44	29.3	128	35.6	
Lower	97	64.7	212	58.9	
Gross type					0.812
Elevated	30	20.0	77	21.4	
Non-elevated	120	80.0	283	78.6	
Depth of invasion					0.420
Mucosa	26	17.3	52	14.4	
Submucosa	124	82.7	308	85.6	
No. of metastatic LN					*0.015*
One	85	56.7	245	68.1	
Two	65	43.3	115	31.9	
Lymphovascular invasion					*<0.001*
Positive	91	60.7	150	41.7	
Negative	59	39.3	210	58.3	
Histologic type					0.656
Differentiated	23	12.5	161	87.5	
Undifferentiated	28	14.1	171	85.9	

*BMI* body mass index, *LN* lymph node, *N* number, *STG* subtotal gastrectomy, *TG* total gastrectomy

*P*-value was italicized when less than 0.05

### Prevalence and predictive factors of tumor recurrence

The median follow-up duration was 78 months (range, 5–216 months). The median time interval from surgical treatment to tumor recurrence was 25.5 months (range, 5–177 months). Results of univariate and multivariate analyses for identifying factors predictive of tumor recurrence are shown in Table 2. Of 510 patients, 38 (7.5%) experienced recurrence while 472 (92.5%) had no recurrence after surgical resection. In univariate analysis, older age (>65 years), male gender, body mass index (BMI) less than 25, elevated gross type, and differentiated histologic type were associated with recurrence. Type of resection, reconstruction method, tumor location, depth of invasion, number of metastatic lymph nodes, number of negative lymph nodes, lymphovascular invasion, and adjuvant chemotherapy were not associated with tumor recurrence. The recurrence rate was 7.3% (11/150) in the adjuvant chemotherapy group, similar to that (7.5%, 27/360) in the surgery-only group. Multivariate analysis showed that older age (>65 years old) and male gender were significant predictive factors for tumor recurrence. The 5-year disease free survival rate was 85.9% in high risk patients satisfying both older age and male gender and 96.3% in other patients (*P* = 0.001). In high risk patients, there was no difference in disease-free survival between the adjuvant chemotherapy group and the surgery-only group either (*P* = 0.511).

**Table 2:** Univariate and multivariate analysis for the identification of predictive factors of recurrence

	Univariate analysis			Multivariate analysis		
	HR	95% CI	*P*	HR	95% CI	*P*
Age						
≤ 65						
> 65	2.625	1.384–4.981	*0.003*	2.541	1.336–4.831	*0.004*
Gender						
Female						
Male	2.401	1.101–5.240	*0.028*	2.217	1.013–4.854	*0.046*
BMI (kg/m2)						
≥ 25						
< 25	2.599	1.086–6.216	*0.032*	2.400	1.001–5.759	0.050
Type of resection						
Subtotal						
Total	0.718	0.221–2.334	0.581			
Tumor location						
Upper						
Middle	0.871	0.193–3.932	0.857			
Lower	1.167	0.276–4.928	0.834			
Gross type						
Non-elevated						
Elevated	2.063	1.054–4.037	*0.035*	1.821	0.929–3.570	0.081
Depth of invasion						
Mucosa						
Submucosa	3.464	0.834–14.394	0.087			
No. of metastatic LN						
One						
Two	1.194	0.623–2.288	0.594			
No. of negative LN	0.996	0.975–1.017	0.697			
Lymphatic invasion						
Negative						
Positive	1.800	0.939–3.454	0.077			
Histologic type						
Undifferentiated						
Differentiated	2.251	1.163–4.356	*0.016*	1.548	0.776–3.087	0.215
Adjuvant CTx						
No						
Yes	0.919	0.455–1.859	0.815	0.952	0.467–1.937	0.891

*BMI* body mass index, *CI* confidence interval, *CTx* chemotherapy, *HR* hazard ratio, *LN* lymph nodes, *LVI* lymphovascular invasion

*P*-value was italicized when less than 0.05

### Patterns of tumor recurrence

As shown in Table 3, 18 patients (47.4%) had tumor recurrence within two years after surgery while 32 patients (84.2%) had tumor recurrence within 5 years after surgery. The most common sites of recurrence were distant lymph nodes including paraaortic, aortocaval, retroperitoneal, retropancreatic, mediastinal, and supraclavicular lymph nodes (11/38, 28.9%). Hematogenous spread (to liver, bone, lungs, and adrenal glands) was present in 23.7% (9/38) of patients. Seven patients (18.4%) showed recurrence in the remnant stomach, two (5.3%) had peritoneal recurrence, and nine (23.7%) showed mixed-type metastasis.

**Table 3:** Patterns of recurrence of pT1N1 gastric cancer

Recurrence				
Pattern	Total (*N* = 38)	Within 2 years (*N* = 18)	2–5 years (*N* = 14)	After 5 years (*N* = 6)
Remnant stomach	7 (A: 2, R: 5)	2 (R: 2)	3 (A: 2, R:1)	2 (R: 2)
Peritoneal	2	2	0	0
Hematogenous	9 (H: 1, P: 1, B: 1)	6 (H: 5, P: 1)	1 (H: 1)	2(H: 1, B: 1)
Distant lymph node	11	3	7	1
Mixed	9	5	3	1

*A* anastomotic site, *B* bone, *H* liver, *P* lung, *R* remnant stomach

## Discussion

The incidence of lymph node metastasis ranges from 10 to 15% in EGC with recurrence rate of 1.4–7.0% [5, 20–23]. In node-positive EGC, recurrence rates are higher (10.6–14.8%) than those of node-negative EGC. Prognosis of EGC after recurrence is very poor. Thus, some investigators have insisted that adjuvant chemotherapy should be considered for node-positive EGC. However, the role of adjuvant chemotherapy for stage I node-positive EGC (pT1N1) remains uncertain. In addition, there is no consensus between the Japanese and NCCN guidelines [3, 4]. Therefore, surgeons sometimes experience confusion on whether to administer adjuvant chemotherapy after surgical treatment in pT1N1 gastric cancer.

Many reports have identified predictive factors for tumor recurrence after surgical treatment in EGC. Although several variables such as lymph node metastasis, lymphovascular invasion, perineural invasion, submucosal invasion, a ratio of metastatic-to-retrieved lymph nodes, elevated gross type, and advanced age have been proposed as prognostic factors for EGC [6, 7, 9–18], few reports are specific for pT1N1 gastric cancer. Moreover, there is no report on the effect of adjuvant chemotherapy on the prognosis of pT1N1 gastric cancer. Accordingly, identifying high risk groups for tumor recurrence in patients with pT1N1 gastric cancer and clarifying the role of adjuvant chemotherapy therein would provide information useful for clinical practice.

In this study, tumor recurrence rate was 7.5% in pT1N1 gastric cancer. Advanced age (>65 years) and male gender were found to be independent predictive factors for tumor recurrence. In addition, adjuvant chemotherapy did not decrease recurrence rate or prolong disease-free survival in pT1N1 gastric cancer in this study. Considering the excellent prognosis of stage I gastric cancer even pT1N1, the limited role of adjuvant chemotherapy was expected to some degree. Therefore, we analyzed risk factors for tumor recurrence and found two independent risk factors (older age and male gender). However, the reason why older age and male gender are independent risk factors for tumor recurrence is currently unclear. This might be related to immunity or comorbidity. However, we could not analyze details about immunity or comorbity because few such data were available. Patients satisfying these two factors showed significantly worse prognosis than those without the two risk factors. Next, the role of adjuvant chemotherapy in these high risk patients was analyzed. We found that, even in high risk patients, adjuvant chemotherapy offered no oncological benefit. According to these results, adjuvant chemotherapy might be unnecessary for pT1N1 gastric cancer patients.

In the present study, the number of metastatic lymph nodes and the presence of lymphovascular invasion were different between the adjuvant chemotherapy group and the surgery only group. These results might reflect the preference of patients and physicians to undergo and administer adjuvant chemotherapy for instances of two metastatic lymph nodes compared to that for one metastatic node. However, since metastatic lymph node number and lymphovascular invasion were not related to tumor recurrence in patients with pT1N1 gastric cancer, these factors should not be used to guide decisions on whether to administer adjuvant chemotherapy.

The results of our study should be interpreted with caution as any retrospective comparison has inherent limitations. Due to excellent prognosis, only a small number of patients experienced tumor recurrence. Although data of patients were collected from three institutions for adequate analysis, small event number might have resulted in some limitations in survival and risk factor analyses. In addition, chemotherapeutic agents were administered heterogeneously because of the development of chemotherapeutic agents and unestablished treatment guidelines. Although the NCCN guideline (Version 2, 2013, Gastric cancer) recommends a few regimens, it could not be used as the standard regimen because there is no evidence for their effectiveness in pT1N1 gastric cancer. In addition, there was no definite regimen recommended during the period of our study. Such heterogenicity in regimens could result in confused outcomes. Although large-scaled prospective randomized trials are needed to clarify this issue, adjuvant chemotherapy might be unnecessary because of excellent prognosis of pT1N1 gastric cancer.

## Conclusions

Adjuvant chemotherapy exhibited no significant benefit for disease-free survival in patients with pT1N1 gastric cancer, suggesting that adjuvant chemotherapy might be unnecessary after curative surgery therein.

## Abbreviations

BMI: Body mass index
EGC: Early gastric cancer
NCCN: National Comprehensive Cancer Network
TS: Titanium silicate
UFT: Tegafur/uracil
